# Effects of Plant-Based Protein Interventions, with and without an Exercise Component, on Body Composition, Strength and Physical Function in Older Adults: A Systematic Review and Meta-Analysis of Randomized Controlled Trials

**DOI:** 10.3390/nu15184060

**Published:** 2023-09-19

**Authors:** Isobel L. Stoodley, Lily M. Williams, Lisa G. Wood

**Affiliations:** 1 Immune Health Program, Hunter Medical Research Institute, New Lambton Heights, NSW 2305, Australia; isobel.stoodley@uon.edu.au (I.L.S.); lily.williams@newcastle.edu.au (L.M.W.); 2 School of Biomedical Science and Pharmacy, University of Newcastle, Callaghan, NSW 2308, Australia

**Keywords:** plant protein, older adults, exercise, animal protein, body composition, muscle mass, strength, physical function

## Abstract

Maintaining muscle mass, strength, and function is crucial for our aging population. Exercise and dietary protein intake are recommended strategies; however, animal proteins have been the most studied. Plant-based protein sources have lower digestibility and incomplete amino acid profiles. However new innovative plant-based proteins and products may have overcome these issues. Therefore, this systematic review aimed to synthesize the current research and evaluate the effects of plant-based protein interventions compared to placebo on body composition, strength, and physical function in older adults (≥60 years old). The secondary aim was whether exercise improved the effectiveness of plant-based protein on these outcomes. Randomized controlled trials up to January 2023 were identified through Medline, EMBASE, CINAHL, and Cochrane Library databases. Studies contained a plant-protein intervention, and assessed body composition, strength, and/or physical function. Thirteen articles were included, all using soy protein (0.6–60 g daily), from 12 weeks to 1 year. Narrative summary reported positive effects on muscle mass over time, with no significant differences compared to controls (no intervention, exercise only, animal protein, or exercise + animal protein interventions). There was limited impact on strength and function. Meta-analysis showed that plant-protein interventions were comparable to controls, in all outcomes. In conclusion, plant-protein interventions improved muscle mass over time, and were comparable to other interventions, warranting further investigation as an anabolic stimulus in this vulnerable population.

## 1. Introduction

Sarcopenia is a syndrome characterised by progressive and generalized loss of skeletal muscle mass, strength, and performance due to aging [1,2,3,4]. It is estimated that a third of the community-based aging population has sarcopenia, though this could be higher in acute or chronic care settings [5]. Development of sarcopenia underpins many health problems in older adults including functional decline, higher rate of falls and hospital admissions, loss of independence, and mortality [1,6,7].

Increasing protein and physical activity, especially resistance training, are the main strategies available for prevention and treatment of the condition [3,5,8]. However, protein-based strategies for sarcopenia are variable, with different types, doses, and timing used with variable effect [9,10,11,12,13,14]. In particular, there is ongoing debate regarding the effectiveness of plant- versus animal-based protein sources in this population.

Plant-based protein sources are a potential alternative to traditional animal-based sources, which require intensive resources to cultivate [15]. Plant protein-rich crops can be produced at lower cost and resource use than animal protein sources, are more easily accessible globally, and produce lower greenhouse gas emissions [15,16,17,18]. Furthermore, plant-based foods can provide additional nutrients such as dietary fibre, polyphenols, and unsaturated fats, while animal-based foods provide minimal quantities of these nutrients, and in fact can provide nutrients with poor impact on health such as saturated fats and sodium [19]. This led the EAT-Lancet Commission to recommend healthy diets with low animal protein sources and increased quantity and diversity of plant-protein sources to not only reduce food waste and provide a sustainable food production model, but also improve global human health, estimated to have the potential to prevent ~11 million deaths per year [20]. 

However, plant-based protein sources have been considered to be less nutritionally effective than animal sources due to reduced digestibility, lower levels of amino acids, and incomplete amino acid profiles [21,22]. Consuming higher amounts of plant-based protein, combining protein sources and use of innovative food manufacturing processes to produce products such as plant-based “mylks” and meat analogues, can provide complete amino acid intakes with improved digestibility that may overcome these limitations [19,21,23,24]. However, in the aging population where protein metabolism is impaired—due to anabolic resistance, reduced muscle protein synthesis, digestive changes, hormonal alterations, chronic inflammation, and insulin resistance—plant-based foods may not provide enough high-quality protein to maintain muscle mass and prevent muscle catabolism [25,26,27]. A recent narrative review suggested that new processing methods may improve nutritional quality of plant proteins for use in older adults [28], while another review suggested the additional nutritional components of plant-based proteins, such as iso-flavones in soy, could be anti-inflammatory [29], reducing the impact of inflammation on muscle protein breakdown to prevent sarcopenia development. It has also been proposed that priming the muscles with exercise prior to plant-based protein intake may overcome the muscle protein synthesis limitations described; meaning plant-proteins could be a good protein source when coupled with exercise, particularly in this vulnerable population [30]. However, previous systematic reviews have found that animal protein is still favourable over plant protein for both younger and older adults [31,32], and the majority of systematic reviews in this space have specifically targeted animal-based protein sources such as whey [33,34,35].

Given the aging Australian and global population, and the increase in quantity and quality of plant-based protein foods available on the market [36,37], a targeted review into the effect of plant-based protein on body composition, strength, and physical function outcomes in older adults is warranted.

The aim of this systematic review was to identify all relevant publications in the area, synthesize the current knowledge, and evaluate the effects of plant-based protein interventions compared to placebo on body composition, strength, and physical function in community-dwelling older adults (≥60 years old). Furthermore, this review aimed to answer the secondary question of whether the addition of an exercise component improved the effect of plant-based protein sources on these outcomes.

## 2. Materials and Methods

### 2.1. Information Source and Search Strategy

This systematic review was conducted according to the Preferred Reporting Items for Systematic Reviews and Meta-analyses (PRISMA) reporting guidelines [38] and registered with PROSPERO (National Institute for Health Research, University of York, York, UK), accessible online at https://www.crd.york.ac.uk/prospero (accessed on 15 June 2023) (CRD42023395191). The electronic databases Embase, CINAHL (Cumulative Index to Nursing and Allied Health Literature), Cochrane Library, and Medline were searched on 29 January 2023 for English language articles from 1947 to the present using predetermined keywords, Medical Subject Heading (MeSH) terms of the National Library of Medicine, and Boolean operators. An example search strategy (the search strategy for Medline database) is presented in Table 1; the search strategies for the other databases are available in Supplementary Tables S1–S3. Briefly, terms were searched relevant to older adults and aging (Search Lines 1–9, combined “OR” for line 10), and terms relevant to plant-based protein (Search Lines 11–16, combined “OR” for line 17). These two search lines were then combined to search relevant articles to both older adults and plant proteins (Search Line 19 “AND”). Finally, animal studies were removed by using the Boolean operator “NOT” in Search Line 20. Additional hand-searching of recently published review articles was conducted.

### 2.2. Eligibility, Inclusion, and Exclusion Criteria

Studies were eligible for inclusion if they met the following requirements: type, randomized controlled trials (RCT); participant, older adults ≥60 years old; investigation, plant-based protein source; control, placebo; outcomes; body composition, strength, or physical function. See Table 2 for the expanded PICO (participant, intervention, comparison, and outcomes) criteria. 

Studies were excluded if they were non-English articles, non-randomized controlled trials, animal studies, in vitro studies, case-control studies, children, and adults <60 years old, participants with cachexia or medical conditions that impacted metabolism (e.g., cancer, heart failure, COPD, critically ill), master athletes, and hospital or residential aged care facility (long-term assistance) settings. Additionally, the plant protein intervention had to clearly be attributed to plant sources (i.e., isolated amino acid studies with no information on origin, were not eligible). 

### 2.3. Data Collection

Following the search, the number of records acquired from each database was recorded, with duplicate studies noted and removed. Abstract and full texts were evaluated using Covidence systematic review software https://support.covidence.org/help/how-can-i-cite-covidence (accessed on 1 June 2023) (Veritas Health Innovation, Melbourne, Australia; www.covidence.org) (accessed on 1 June 2023). Two reviewers (I.L.S. and L.M.W.) assessed the studies on title, abstract, keywords, and MeSH terms using the inclusion and exclusion criteria outlined above. Irrelevant studies were noted and then removed. Full texts of remaining studies, including studies with unclear relevance, were retrieved, and assessed for relevance by two reviewers (I.L.S. and L.M.W.), according to the inclusion and exclusion criteria. If there was any disagreement between the two reviewers regarding the relevance of a study, a third independent reviewer was involved (L.G.W.), and a consensus was reached.

### 2.4. Risk of Bias Assessment

The remaining studies were assessed for quality by two reviewers (I.L.S. and L.M.W.), using a standardized critical appraisal checklist designed by the American Dietetic Association (ADA) [39]. This tool assessed the reliability, validity, and generalizability of the included studies. Studies appraised to be of poor quality (response to ≥6 validity questions “no”) were excluded.

### 2.5. Data Extraction

After eliminating duplicate, irrelevant, and negative quality studies, study details were extracted and recorded using a standardized data template in Covidence. Data extracted included: author, publication year, country, baseline characteristics (age, sex, sample size, setting, inclusion and exclusion criteria), intervention details (type, dose, frequency, and timing) and outcomes of interest (body composition, strength, physical function). Studies were reported qualitatively in a table format, with columns for country, age, inclusion and exclusion criteria, sample size (by sex), study quality as determined by the ADA checklist, details of the intervention, details of control, and the effect of the intervention on outcomes of interest. 

### 2.6. Meta-Analysis

Meta-analysis was performed using Review Manager (RevMan, Version 5.3. The Cochrane Collaboration, (2014). https://documentation.cochrane.org/revman-kb/cite-revman-web-in-a-reference-list-110242006.html accessed on 18 June 2023). A random-effects model was used to determine the overall effect size of the intervention, to account for potential heterogeneity between studies [40]. If reported, difference between pre-intervention and post-intervention means (mean*_diff_*) and standard deviations (*SD_diff_*) were retrieved. Where outcomes were reported as 95% confidence intervals or standard error of mean (SEM), they were converted to *SD* [41]. If only pre-intervention and post-intervention means (and *SD*s) were reported, mean*_diff_* and *SD_diff_* were calculated for both intervention and control group, following the Cochrane Handbook for Systematic Reviews of Interventions [41]. For calculating *SD_diff_*, a correlation coefficient (*corr*) for each outcome was calculated (see below) from studies within the review which provided sufficient detail. Where no included study reported sufficient details, the *corr* was calculated from an external study with similar design and methodology:

*corr* = (*SD_pre_*2 + *SD_post_*2 − *SD_diff_*2) (2 × *SD_pre_* × *SD_post_*)

Then, *SD_diff_* was calculated as below:

*SD_diff_* = √(*SD_pre_*2 + *SD_post_*2 − 2 × *corr* × *SD_pre_* × *SD_post_*)

where *SD_post_* is the *SD* of the post-intervention mean and *SD_pre_* is the *SD* of the pre-intervention mean, as stipulated in the Cochrane Handbook for Systematic Reviews of Interventions Version 6.2, Chapter 6: Choosing effect measures and computing estimates of effect [41]. 

When data was not reported or reported in a way that could not be synthesized from the above methods, the corresponding author was contacted to request the required data. If the author did not respond, the study was excluded from the meta-analysis.

Standardized mean difference (SMD) was reported when different scales were used to measure the same outcome (e.g., knee extension strength is frequently measured using Newton meters or kilograms, or when lean muscle mass was measured with either dual energy X-ray absorptiometry—DEXA, or bioelectrical impedance analysis—BIA). Effect sizes were determined using Cohen’s method—an SMD between 0.2–0.5 was considered a small effect, between 0.5–0.8 a moderate effect, and >0.8 a large effect [42]. Where the same methodology and outcome unit were used, mean difference (MD) was reported.

Heterogeneity was investigated using the χ^2^ test (*p* < 0.1 considered to indicate significant heterogeneity) and *I*^2^ parameter (30–60% indicating moderate, 50–90% indicating substantial, and 75–100% indicating considerable heterogeneity) [41]. 

Sensitivity analysis was performed using the leave-one-out method in RevMan [43]. This involved removing individual studies one at a time for all variables to determine if there was an effect on the meta-analysis outcome. Funnel plots were visually assessed for asymmetry to determine if publication bias was probable. 

To answer the secondary question, sub-group analysis was performed where appropriate. Studies were separated depending on whether an exercise component was present. Sub-group analysis was conducted when a minimum of three studies could be included in a group. Groups with <3 studies were either removed from the analysis (if there were >2 groups available), or sub-group analysis was not performed.

Where multiple comparators were available, the closest to a control (no additional interventions) was used in the main analysis. Separate analyses were conducted for trials with similar alternative comparisons (versus animal protein, containing an exercise intervention).

## 3. Results

### 3.1. Study Selection

**Figure 1:**
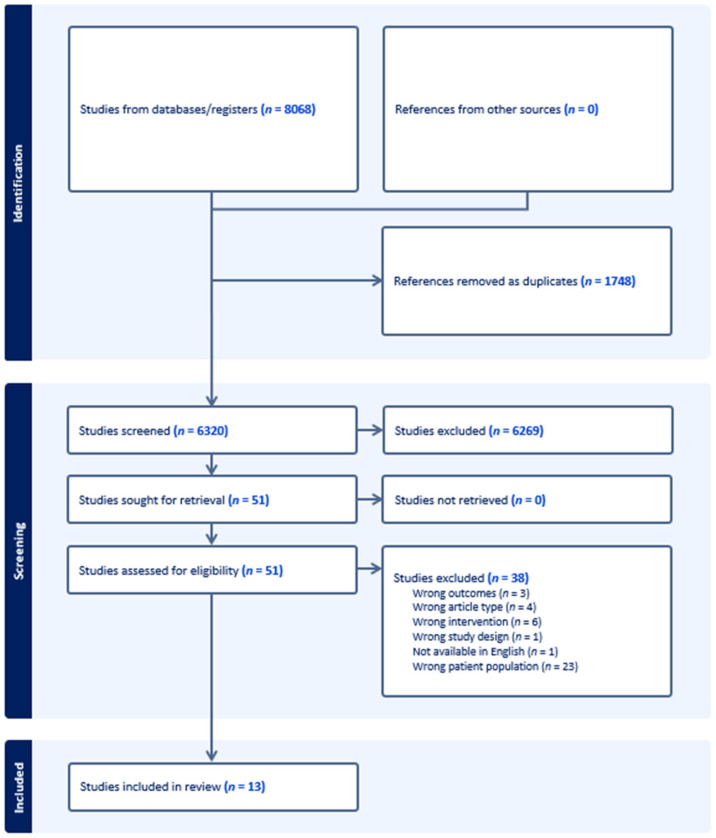
Preferred Reporting Items for Systematic Reviews and Meta-Analyses (PRISMA) flow diagram.

The search identified 8068 studies from four databases (Medline *n* = 2541, Embase *n* = 2523, Cochrane Library *n* = 2270, CINAHL *n* = 734), of which 1748 were removed as duplicates (Figure 1). Handsearching did not reveal any further studies. The remaining 6320 studies were assessed for relevancy via title and abstract, of which 50 studies were eligible for full text assessment. Following full text review, 13 studies were eligible for inclusion. According to the ADA checklist, no studies were removed due to poor methodological quality. Two articles had neutral quality (Kok [44] and Haub [45]), however, both studies were reported in additional papers included in this review (Kreijkamp-Kaspers [46] and Haub [47] respectively) with positive quality. For simplicity, each of these studies is referred to by referencing the positive quality paper published.

### 3.2. Study Characteristics

The included study characteristics are reported in Table 3. The studies were conducted in United States (*n* = 3, [47,48,49]), Japan (*n* = 3, [50,51,52]), Iran (*n* = 2, [53,54]), Netherlands (*n* = 1, [46]), China (*n* = 1, [55]), and Brazil (*n* = 1, [56]). In total, there were 806 participants included from studies published between 2002–2022. The majority of studies excluded conditions that would affect muscle mass such as severe or uncontrolled cardiovascular, respiratory, muscular, metabolic, inflammatory, renal, hepatic, or bone diseases. Other common exclusions were medication for the above conditions, protein supplementation, smoking, and contraindication to the study intervention (e.g., soy/milk allergies, exercise restrictions).

### 3.3. Intervention Characteristics

Intervention details are summarized in Table 4. All studies used soy as the protein source. The study intervention duration varied between 12 weeks to 1 year. The overall daily dose of protein varied from 0.6 g to 60 g. Most studies provided protein supplementation daily, except for Imaoka et al. [50,51] which provided one 4.4 g dose of soy peptide drink once per week. Six studies included an exercise component of either resistance training (RT), aerobic training or both [47,50,51,52,54,56]. Bijeh et al. [54] was the only study to include a soy only arm, and a soy + RT arm, which could be compared separately to a control (no intervention) and RT only arm, respectively. Comparisons included animal protein [46,47,48,49,52,54,55,56], no intervention control [53,55], and exercise only controls [50,51,54,56]. Bakhtiari et al. [53] provided two soy arms (soy nut or textured soy protein) compared to no intervention control, while Beavers et al. [49] was a dedicated weight loss study with both plant-protein and animal-protein (control) interventions in a planned calorie deficit.

**Table 3:** Included study characteristics, eligibility criteria, sample size, and quality assessment.

Author, Year Published	Country	Study Design	Age	Inclusion Criteria	Exclusion Criteria	Sample Size and Sex	Quality
Bakhtiari [53]	Iran	Non-blinded RCT	60–70	Metabolic Syndrome defined as ≥3 of following: waist circumference >80 cm; serum HDL-C < 50 mg/dL; triglyceride ≥150 mg/dL; fasting blood glucose ≥100 mg/dL; and systolic blood pressure ≥130 mmHg and diastolic ≥85 mmHg)	Medication for diabetes, hypertension, hyperlipidaemia; estrogen therapy; soy consumption; history of CVD; thyroid condition; kidney or liver conditions; infectious disease; cancer; vegetarian; smokers or soy allergy	75♀	+
Beavers [49]	United States	Single-blind RCT	60–79	BMI ≥ 27 kg/m^2^, waist circumference ≥102 cm ♂ and 88 cm ♀, willing to consume prepared meals and meal replacement products; and no contraindications for participation in a weight loss program	Weight change (±5%) in the past 6 months; body mass >136 kg; regular smoker; alcohol or substance abuse ≤2 years; insulin-dependent or uncontrolled diabetes; abnormal kidney or liver function; past or current ischemic heart disease; uncontrolled blood pressure (>160/90 mmHg), pulmonary disease; thyroid disease; known significant haematological disease; cancer requiring treatment in past year, or life expectancy <2 years; and regular use of any medications that could influence study variables (growth/steroid hormones, including estrogen replacements, thiazolidinediones, statins, regular anti-inflammatory medications, blood thinners, or weight loss medications)	21♀ 3♂	+
Bijeh [54]	Iran	Double-blind RCT	60–80	Physically independent	CVD, neurological, respiratory, muscular, metabolic, inflammatory, bone problems, joints, and movement disorders; consuming nutritional supplements; consuming drugs affecting muscle metabolism; consuming alcohol or smoking ≥1 year; soy milk allergy/sensitivity, and history of regular physical activity ≥1 year.	60♂	+
Haub [45,47]	United States	Non-blinded RCT	65 ± 5		Medical conditions that might place them at risk if they participated in the study	21♂	+ (2002), Ø (2005)
Imaoka [51]	Japan	Non-blinded RCT	≥60	Community dwelling, physically independent	Collagen disease; depression; CVD; medical contraindications to exercise; or Parkinson’s disease	61♀ 13♂	+
Imaoka [50]	Japan	Non-blinded RCT	≥60	Community dwelling, physically independent	Doctors’ orders to stop exercise, medicalcontraindications to exercise; dementia	59♀ 13♂	+
Kenny [48]	United States	Double-blind RCT	≥60		Diseases that could affect bone metabolism (Paget’s disease, thyroid conditions, osteomalacia, multiple myeloma); cancer ≤5 years; calcitonin, calcitriol, heparin, phenytoin, or phenobarbital use ≤2 years; bisphosphonates or corticosteroid use ≥6 months; methotrexate or fluoride use; creatinine clearance <50 mL/min; liver disease; history of hip fracture; known vertebral fracture ≤1 year; and vegan	131♀	+
Kok [44] Kreijkamp-Kaspers [46]	Netherlands	Double-blind RCT	60–75	Normal mammography ≤1 year	Liver disease; renal disease; thrombosis; malignant disease; hormone replacement therapy ≤6 months; soy or casein allergy; lactose intolerance; endometrium thickness over 4 mm	202♀	Ø (2005), + (2004)
Li [55]	China	Double-blind RCT	≥65	Low appendicular skeletal muscle mass index (♂ < 7.0 kg/m^2^, ♀ < 5.4 kg/m^2^)	Diseases with impaired movement (stroke, fracture, and arthritis); kidney disease; nervous system disease; joint replacement; musculoskeletal injuries; whey or soy allergy; supplement use ≤1 year; and unwillingness to adhere to the study protocol	62♀ 61♂	+
Matsuda [52]	Japan	Single-blind RCT	65–80	HbA1c 6.5 to <8.5%; HbA1c change of ≤1.0% ≤6 months	Diabetes other than T2DM; receiving insulin, growth hormone, glucocorticoids, or anabolic steroids; eGFR < 30 mL/min/1.73 m^2^; proliferative retinopathy; contraindication to exercise due to bone and joint disease; current treatment for malignancy	13♀ 23♂	+
Roschel [56]	Brazil	Double-blind RCT	>65 years old	Pre-frail or frail based on Fried’s criteria—unintentional weight loss, weakness, self-reported exhaustion, slow walking speed, and low physical activity	Insulin or steroid-based drugs; protein supplements; caloric or food restriction; resistance training; untreated chronic disease or any musculoskeletal condition contraindicated for exercising	60♀	+

Abbreviations: BMI, body mass index; CVD, cardiovascular disease; eGFR, estimated glomerular filtration rate; HbA1c; glycated haemoglobin; HDL-C, high density lipoprotein-cholesterol; RCT, randomized controlled trial; T2DM, type II diabetes mellitus; ♀ female; ♂ male; +, positive quality as per the American Dietetic Association (ADA) standardized critical appraisal checklist; Ø, neutral quality as per the ADA standardized critical appraisal checklist.

### 3.4. Outcomes of Plant Protein Interventions on Body Composition, Strength, and Physical Function

The narrative summaries of plant protein interventions on outcomes of body composition, strength, and physical function are described in Table 4. Many studies reported an overall positive effect of the soy interventions over time, with no significant differences between groups reported with comparison interventions of animal protein, exercise only, exercise + animal protein, or no intervention [47,49,50,51,52,53,55,56]. The benefits of plant based protein interventions were strongest for lean muscle mass maintenance and accrual as measured by DEXA or BIA [50,51,53,55,56], with no effect seen in any intervention group for bone mineral density (DEXA) [46,48]. Lean mass and fat mass (DEXA) decreased in both groups (weight loss study, plant versus animal protein supplements) in Beavers et al. [49], while Bijeh et al. [54] reported the strongest interaction for muscle mass (BIA) accrual in the soy + RT group. Two studies reported no changes in body composition outcomes for either the plant protein group or the comparison group (exercise + animal protein) [47,52].

The effect of plant protein interventions for strength and physical function varied between studies. Grip or knee strength (as measured by dynamometry) improved in four studies [45,51,52,54] though only Bijeh et al. [54] reported a group effect, with soy + RT performing the best. Five studies [46,49,50,55,56] reported no change or a decrease in strength over the intervention period. Similarly, physical function did not improve in three studies [49,50,56] while three studies reported improvements over time but no difference in either gait speed, SPPB, or TUG, when compared to animal protein, exercise only, or no intervention [46,51,55].

### 3.5. Meta-Analyses for the Effect of Plant Protein Interventions on Body Composition, Strength, and Physical Function

Meta-analyses were performed to determine the effect of plant protein interventions on body composition, strength, and physical function outcomes. Bone mineral density, thirty second sit-to-stand, chair stand test, and timed up-and-go outcomes could not be analysed as <3 studies reported these outcomes.

**Figure 2:**
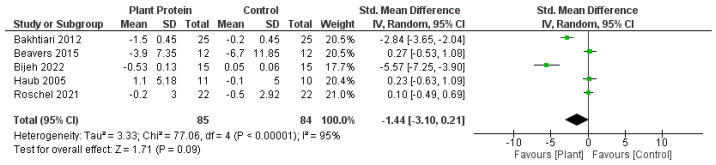
Forest plot of randomized controlled studies examining the effect of plant protein interventions on fat mass. Individual study effect estimates (green boxes) and the pooled effect estimate (diamond) are shown. Values are standardized mean differences with 95% confidence intervals (CI) determined using generic inverse-variance random-effects models. Heterogeneity was quantified by *I*^2^ at a significance of *p* < 0.10 [47,49,53,54,56].

**Figure 3:**
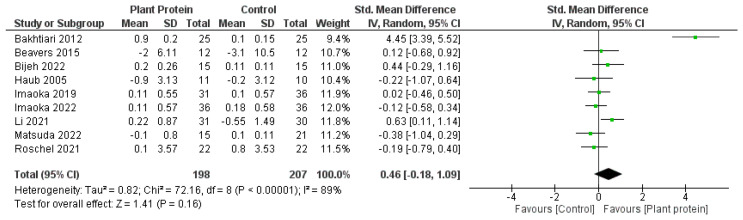
Forest plot of randomized controlled studies examining the effect of plant protein interventions on lean muscle mass. Individual study effect estimates (green boxes) and the pooled effect estimate (diamond) are shown. Values are standardized mean differences with 95% confidence intervals (CI) determined using generic inverse-variance random-effects models. Heterogeneity was quantified by *I*^2^ at a significance of *p* < 0.10 [47,49,50,51,52,53,54,55,56].

**Figure 4:**
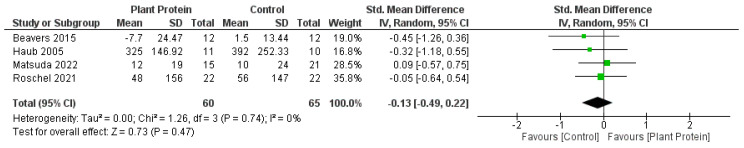
Forest plot of randomized controlled studies examining the effect of plant protein interventions on knee extension strength. Individual study effect estimates (green boxes) and the pooled effect estimate (diamond) are shown. Values are standardized mean differences with 95% confidence intervals (CI) determined using generic inverse-variance random-effects models. Heterogeneity was quantified by *I*^2^ at a significance of *p* < 0.10 [47,49,52,56].

**Figure 5:**
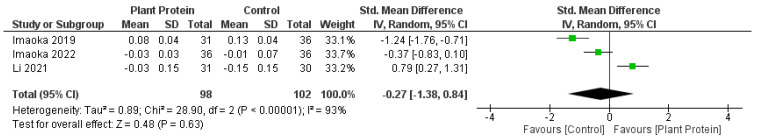
Forest plot of randomized controlled studies examining the effect of plant protein interventions on gait speed. Individual study effect estimates (green boxes) and the pooled effect estimate (diamond) are shown. Values are standardized mean differences with 95% confidence intervals (CI) determined using generic inverse-variance random-effects models. Heterogeneity was quantified by *I*^2^ at a significance of *p* < 0.10 [50,51,55].

Overall, there was no significant difference between plant protein interventions compared to animal protein, exercise only, exercise + animal protein, or no intervention control in any outcome. Fat mass loss favoured plant protein interventions compared to animal protein, exercise only, exercise + animal protein, or no intervention controls; however it was not statistically significant (SMD—1.44, 95%CI—3.10, 0.21, *p* = 0.09) (Figure 2). Lean muscle mass accrual also favoured plant protein interventions compared to controls of either animal protein, exercise only, exercise + animal protein, or no intervention; however, it was also not significant (SMD—0.46, 95%CI—0.18, 1.09, *p* = 0.16) (Figure 3). Knee extension strength and gait speed appeared to favour the control groups of animal protein or exercise + animal protein rather than plant protein interventions (Figure 4 and Figure 5).

### 3.6. Subgroup Meta-Analyses

Given the types of comparison interventions available, two subgroups emerged: The planned comparison between interventions with or without an exercise component, and given the number of studies, a specific comparison to studies with animal protein groups. These sub-group analyses could only be performed for lean muscle mass to ensure at least three studies in each sub-group.

**Figure 6:**
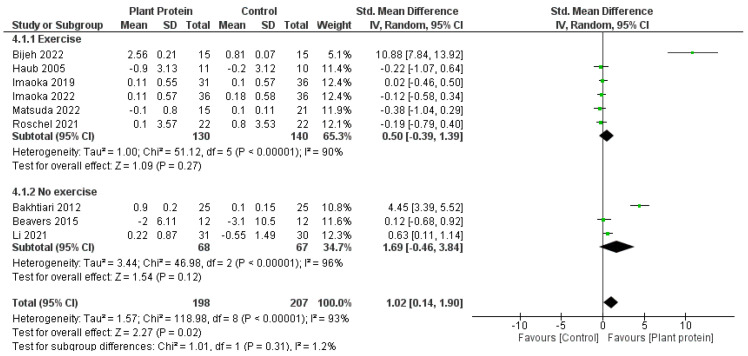
Forest plot of randomized controlled studies examining the effect of plant protein interventions on lean muscle mass, sub-grouped by exercise component. Individual study effect estimates (green boxes) and the pooled effect estimate (diamond) are shown. Values are standardized mean differences with 95% confidence intervals (CI) determined using generic inverse-variance random-effects models. Heterogeneity was quantified by *I*^2^ at a significance of *p* < 0.10 [47,49,50,51,52,53,54,55,56].

When comparison studies which included an exercise component to those without (Figure 6), the meta-analysis favoured the non-exercise studies (overall effect *p* = 0.02, subgroup differences *p* = 0.31). Interestingly, when including the exercise arms (as opposed to the initial analysis which compared the control arms where multiple comparison arms existed), the overall effect for lean muscle mass accrual significantly favoured the plant protein groups (SMD 1.02, 95%CI 0.14, 1.90, *p* = 0.02).

**Figure 7:**
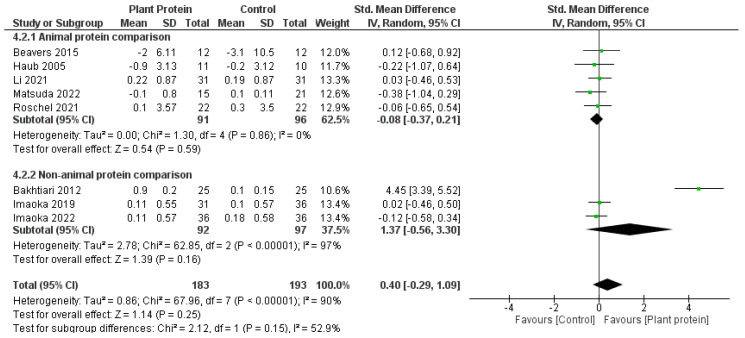
Forest plot of randomized controlled studies examining the effect of plant protein interventions on lean muscle mass, sub-grouped by protein comparison component. Individual study effect estimates (green boxes) and the pooled effect estimate (diamond) are shown. Values are standardized mean differences with 95% confidence intervals (CI) determined using generic inverse-variance random-effects models. Heterogeneity was quantified by *I*^2^ at a significance of *p* < 0.10 [47,49,50,51,52,53,54,55,56].

When comparing studies which included an animal protein comparator to those without (Figure 7), the meta-analysis favoured the animal protein comparator, compared to the plant protein group (animal protein subgroup SMD −0.08, 95%CI −0.37 to 0.21, *p* = 0.59; versus non-animal protein subgroup SMD 1.37, 95%CI −0.56 to 3.30, *p* = 0.16; subgroup differences 0.15). Interestingly, when the comparator was a non-animal protein intervention, the results favoured the plant protein intervention group, however this was not statistically significant. 

### 3.7. Sensitivity Analysis and Publication Bias

When completing the sensitivity analysis using the leave-one-out process, no meta-analyses changed. When removing Bakhtiari et al. [53] from the lean muscle mass outcome, Bijeh et al. [54] from the grip strength outcome, or Li et al. [55] from the gait speed or SPPB outcomes, the SMD moved more to favour the control group, however the *p*-value did not change. These studies were also the outliers when analysing the funnel plots. 

## 4. Discussion

This systematic review aimed to summarize the current research for plant-based protein interventions for improving body composition, strength, and physical function in older adults. This review found that there were positive effects reported on lean muscle mass accrual and strength over time. Further there were no significant differences between plant-based protein interventions and control interventions, which included animal protein, exercise only, exercise + animal protein, or no intervention controls. Meta-analyses indicated plant proteins were comparable to control interventions. Knee extension strength favoured animal protein or exercise + animal protein controls, however this was not statistically significant. Subgroup meta-analysis did not report any further significant differences, though lean muscle mass accrual favoured plant protein interventions when there was no exercise component, or the comparison was not animal protein. Overall, this review found positive effects for plant-based protein interventions in older adults, which warrant further investigation in this population.

### 4.1. Plant-Based Proteins and Body Composition Outcomes

Lean muscle mass was one outcome that indicated a positive effect of plant protein supplementation, both in narrative summary and meta-analysis. However, when adjusting based on comparators, plant protein interventions were only favoured compared to non-animal protein controls. This is not surprising, with many studies and reviews previously documenting that animal protein sources have the strongest evidence for improving muscle mass by stimulating muscle protein synthesis pathways, such as mammalian target of rapamycin (mTOR) in older adults [21,31,32]. However, it does highlight there are some benefits for plant-based protein options in older adults, as they still performed better than comparators such as exercise alone, or no intervention. With growing vegetarian and vegan populations [57], reviews such as ours highlight that plant-based protein sources may still provide benefit in older adults who experience impaired anabolic metabolism [27,58]. 

In addition to improving muscle mass, this review found that plant proteins favoured fat mass loss, indicating that plant proteins may be beneficial for overall body composition. As we age, fat mass increases, while lean muscle mass peaks in mid age [59,60]. This is concerning for older adults, as higher fat mass is associated with greater muscle mass decline, insulin resistance, and increased morbidity and mortality risk [61,62]. This may be due to inflammaging—the chronic, low-grade inflammation that develops with aging [26]. Increased adipose tissue, particularly centrally, increases the pro-inflammatory state, by producing adipokines that mediate inflammation such as adiponectin and leptin [63]. Therefore, it is of great interest to find lifestyle interventions, such as increasing plant protein intake, that may reduce fat mass in a high-risk population such as older adults. While our review indicates plant proteins may be beneficial for fat loss, this may not be different from animal-based protein sources [64]. High protein diets compared to low protein diets increase thermogenesis and satiety, which has increased their utility in the weight loss space [65]. While this review did not investigate biochemical markers of inflammation, our results on total fat mass loss warrant further investigation on these outcomes related to healthy aging.

This review does not support the use of plant protein sources to improve bone density in older adults. While only two studies included in this review measured bone density, other studies and reviews do not indicate that plant or animal protein sources are useful for improving bone density for adults > 18 years old [66,67,68]. Itkonen et al. [69] reported that plant proteins increased markers of bone resorption, and this was proposed due to the lower vitamin D and calcium contents in these diets. While isoflavones have been suggested to be positive for bone outcomes in post-menopausal women [70], the overall consensus is that soy is neutral on bone-related outcomes [71]. 

### 4.2. Plant-Based Proteins and Strength and Physical Function Outcomes

Similar to lean muscle mass, there appeared to be no difference of plant-protein supplementation compared to control on outcomes related to strength and physical function. Grip or knee extension strength improved in four studies supplementing with plant proteins, while physical function as assessed by gait speed, SPPB or TUG improved in three studies. While meta-analysis was not possible for these outcomes due to the small numbers, the narrative review indicates that plant-based proteins performed similarly to other comparators including animal protein or exercise only, and could be used in this population to prevent strength and functional decline. Beyond supplying amino acids for building muscle, the additional nutritive components of plant-based sources of protein such as vitamins, minerals, antioxidants and fibre have been proposed to impact muscle health by reducing inflammation and ameliorating the negative effect of reactive oxygen species on muscle tissue, allowing improvement of muscle strength and function [23,72,73]. However a recent review by Coelho-Júnior [74] reported that the source of protein had no clear association with physical function, and instead total protein was associated with better muscle performance. This is akin our review, which found that plant-based sources appeared to generally perform similarly to other interventions such as animal-based protein and exercise interventions. While plant-based diets may be beneficial for morbidity and mortality in conditions such as ischemic heart disease and cancer [75], their utility for muscle health and conditions such as sarcopenia appear promising. They warrant further investigation as they did not appear to be inferior compared to other interventions in our review.

### 4.3. Plant-Based Proteins in Combination with Exercise

Interestingly when comparing studies with exercise components, plant-based proteins performed better without an exercise component. This was surprising as exercise is known to stimulate anabolism [9,12,76,77] and it is proposed that the addition of exercise primes the muscles and overcomes the limitations of plant proteins such as incomplete amino acids [30]. However, another hypothesis is that additional nutritional components of plant-based proteins, such as iso-flavones in soy, could be anti-inflammatory [29], reducing the impact of inflammaging (low grade chronic inflammation that occurs with aging [26]) on muscle protein breakdown and improving overall muscle protein synthesis. Overall, our review suggests that exercise does not improve the action of plant proteins.

### 4.4. Plant-Based Proteins and Dietary Patterns

Of note, there was an emerging number of studies from Japan identified in this review. Japan is one of the highest consumers of soy globally, however this has started to fall in recent years [36,78]. Japan also has one of the longest life expectancies (≥80 years old) and the greatest number of centenarians [79]. The Japanese/Okinawan diet has been proposed as a key determinant of healthy aging in this population; however there are other components of this dietary pattern beyond high soy consumption that could explain this relationship. For example, the Japanese/Okinawan diet is high in fish (omega-3 polyunsaturated fatty acids), vegetables (antioxidants, fibre), green tea (antioxidants, polyphenols) and contains the cultural practice of only eating until 80% full (“hara hachi bu”, calorie restriction), that may also play an interconnected role [73]. The results from our systematic review suggest that soy consumption may be beneficial in this population for outcomes related to healthy aging, however other reviews on the additional components of the Japanese/Okinawan diet have also reported beneficial effects on these same outcomes [80,81,82,83]. While our current review cannot comment on the additional components of the Japanese/Okinawan diet, it is of note that dietary patterns are a growing research interest area in the sarcopenia space, and so far research indicates the dietary patterns such as the Japanese/Okinawan diet or the Mediterranean diet are beneficial for healthy aging, compared to Western dietary patterns which appear more harmful [73,84,85]. As of 2022, the United States Department of Agriculture concluded there was no significant benefit of any particular dietary pattern for sarcopenia [86], however this may change as future research moves from specific nutrients such as protein to overall dietary patterns.

### 4.5. Strengths and Limitations

One of the main limitations of this review was that only soy protein sources were identified in our search. While soy has the longest history of use as a plant-based protein source [36], there are many emerging plant-based protein sources such as other legumes (chickpea, lentil, pea), cereals and grains, nuts and seeds, algae, and microbial protein sources (such as fungi, microalgae, and bacteria) [16] that are part of the growing plant protein industry in Australia and globally [37]. We had hoped to include many varied sources in this review, in order to conduct a holistic approach of the plant-based protein market. Given that only soy products were represented in this review, it highlights an emerging space for this vulnerable population. More studies are needed in the older adult population (≥60 years old) to determine the efficacy of alternative plant-based protein sources for sarcopenia-related outcomes. 

A strength of our review was restricting the search to only RCTs, which provide high quality evidence. Another strength was designing the search to capture a variety of plant-based protein sources; a limitation was that only soy sources met our other criteria such as age ranges and outcomes. Therefore, our review failed to capture the effect of other sources, such as pea protein. Another limitation was that our meta-analyses reported high heterogeneity which was not explained by our sub-groups examining exercise inclusion and animal protein comparison. Factors that could explain this heterogeneity include dose of protein, study duration, and study population, which all varied considerably between our included studies. Additionally, none of the included studies accounted for baseline protein intake when assessing participant eligibility, therefore the included participants could be already consuming adequate protein, minimizing the effect gained by supplementing with plant-based sources. Finally, our systematic review is susceptible to the general limitations of RCTs and systematic reviews, including selection bias, publication bias, and attrition bias [87,88], which despite attempting to account for by using the ADA standardized critical appraisal checklist, cannot be avoided completely. Many studies excluded in our review were for “post-menopausal” women, which could refer to anyone ≥45 years, so while our criteria of ≥60 years old was aimed to capture “older adults” it may have been overly restrictive. Nonetheless, we highlighted that interventions specifically targeting these older adults (≥60 years old) with new plant proteins such as pea, are needed to determine efficacy. Another limitation was the mixed populations of the studies included, which prevented us from comparing males and females separately.

## 5. Conclusions

Overall, our review indicated that plant proteins may be beneficial in older adults to maintain muscle mass. Plant proteins were comparable to the control interventions including animal protein, exercise only, exercise + animal protein, or no intervention controls. Future research should target older adults (≥60 years old), experiment with newer plant proteins such as pea and microalgae, and specifically report outcomes in males and females separately to tease out sex differences between protein sources.

## Figures and Tables

**Table 1:** Example search strategy (Medline database).

Search Line	Search Terms
1	SARCOPENIA/
2	AGED/
3	AGING/
4	“Aged, 80 and over”
5	FRAIL ELDERLY/
6	FRAILTY/
7	(dynapen* OR anabolic resistance).mp.
8	(older adult OR older OR senior OR elder OR elderly OR ?enarian OR geriatric).mp.
9	(sarcop?eni* OR aged OR aging OR frail elderly OR frailty).tw.
10	1 OR 2 OR 3 OR 4 OR 5 OR 6 OR 7 OR 8 OR 9
11	Plant Proteins/
12	Vegetable Proteins/
13	Soybean Proteins/
14	(plant protein* OR vegetable protein* OR soybean protein* OR plant-base*).tw.
15	Soy milk/
16	soy milk.tw.
17	11 OR 12 OR 13 OR 14 OR 15 OR 16
18	animals/NOT (humans/AND animals/)
19	10 AND 17
20	19 AND 18

Wildcards (? Or *) used to expand search terms to cover alternate spellings or variations on a root word. E.g., dynapen* will search dynapenia, dynapenic, etc.; and sarcop?enia will search sarcopenia or sarcopaenia.

**Table 2:** Participants, intervention, controls, and outcomes (PICO) table.

PICO Component	Inclusion Criteria
Participants	Community dwelling older adults of either sex, ≥60 years old
Intervention	Plant-based protein interventions, with or without an exercise component, at least 6 weeks in duration. Supplements or whole foods that can clearly be attributed to plant sources can be included. Any protein sources of unclear origin (e.g., isolated amino acids) will be excluded. Any exercise intervention can be included (aerobic training, resistance training, combined, etc.) Setting: gym facility or home-based interventions, with any supervision type (face to face training, online supervision, no supervision at all, etc.)
Controls	Placebo interventions (with or without an exercise component). This may include non-protein dietary interventions or animal-based protein interventions. Exercise only controls.
Outcomes	Body composition: lean muscle mass, appendicular muscle mass, fat mass, bone density or bone content (e.g., dual energy X-ray absorptiometry—DEXA, bioelectrical impedance analysis—BIA, computer tomography—CT, or air displacement plethysmography—BodPod). Strength: Grip strength (e.g., dynamometer), knee extension strength (e.g., dynamometer), thirty second sit-to-stand, 5 chair stand test. Function: gait speed (e.g., 3–10 m walk tests, 400 m walk test), short physical performance battery, timed up and go.

**Table 4:** Included study intervention characteristics.

Study	Duration	Plant Protein Intervention Protein Type Protein Dose/Serve Frequency Total Daily Dose * Exercise Component	Comparison Protein Type Protein Dose/Serve Frequency Total Daily Dose * Exercise Component	Narrative Summary Body Composition	Narrative Summary Strength	Narrative Summary Physical Function
Bakhtiari [53]	12 weeks	Soy nut 13.8 g 1/day **13.8 g/day**	(1) Control (nothing) (2) Textured soy protein **18.2 g/day**	Mild positive effect of both soy groups but not significant between groups. Lean mass (BIA) increased in soy nut group compared to control, and both soy groups decreased fat mass (BIA) over time.		
Beavers [49]	12 weeks	Soy protein meal replacement products 11–15 g 4/day **44–60 g/day**	(1) Non-soy (whey and egg) meal replacement products 11–15 g 4/day **44–60 g/day**	Lean mass, fat mass (DEXA) reduced in both groups, no between group interactions.	Knee extensor strength (isokinetic dynamometer) significantly reduced in both groups. Grip strength (dynamometer) did not change over time in either group.	Gait speed (400 m walk time) and SPPB did not change over time in either group.
Bijeh [54]	12 weeks	Soy milk 6.75 g 1/day **6.75 g/day** RT 3/week	(1) RT 3/week (2) RT + Soy milk **6.75 g/day** RT 3/week (3) Control	Muscle mass (BIA) increased in RT and RT+ soy group over time. Significant group and time effect interaction for fat mass (BIA). No change in control group over time.	Significant group and time effect interaction for grip strength (dynamometer), with RT + soy milk group performing best.	
Haub [45,47]	12 weeks	Textured vegetable protein products (soy) **0.6 g/kg/day** RT 3/week	(1) Beef foods + RT **0.6 g/kg/day** RT 3/week	No overall response in either group for muscle mass or fat mass (BodPod). Mid-thigh muscle (CT) increased in both groups over time. No significant differences between groups.	Knee extension strength (pneumatically adjusted leg extension machine) increased in both groups, no significant differences between groups.	
Imaoka [51]	3 months	Soy peptide drink 4.4 g 1/week **0.6 g/day** AE 1/week	(1) AE 1/week	Skeletal muscle (BIA) improved in both groups over time, with no group interaction.	Grip strength (dynamometer) improved in both groups over time, with no group interaction.	Gait speed (2.4 m walk test) improved in both groups over time, with no group interaction.
Imaoka [50]	3 months	Soy peptide drink 4.4 g 1/week **0.6 g/day** AE 1/week	(1) AE 1/week	Skeletal muscle (BIA) improved in both groups over time, with no group interaction.	No group or time effects for grip strength (dynamometer).	No time or group effects for gait speed (2.4 m walk test).
Kenny [48]	1 year	Soy protein isolate (placebo isoflavone tablets) 18 g 1/day **18 g/day**	(1) Control protein: casein (50%), whey (25%) and egg white (25%) isolate + placebo isoflavone tablets **18 g/day** (2) Soy protein + isoflavone tablets (3) Control protein + isoflavone tablets	No group or time effects for BMD (DEXA).		
Kok [44] Kreijkamp-Kaspers [46]	1 year	Soy protein 25.6 g 1/day **25.6 g/day**	(1) Milk protein 25.6 g 1/day **25.6 g/day**	Both groups decreased BMD (DEXA) after a year. Hip (intertrochanter region), had significant difference between groups with increase in soy group, reduction in control group. No other differences in other hip regions or spine.	No significant difference between groups, however hand grip (dynamometer) not measured at baseline. Results adjusted by baseline age, BMI, past use of HRT, postmenopausal years, fertile years, and height did not impact results.	Slight increases in SPPB score in both groups, no significant differences between groups.
Li [55]	6 months	Soy protein 8.80 g 2/day **17.6 g/day**	(1) Whey Protein 7.89 g 2/day **15.78 g/day** (2) Combined whey-soy blend 8.39 g 2/day **16.78 g/day** (3) control	ASMMI, lean mass in legs (DEXA) maintained in the supplement groups compared to control which decreased from baseline. No significant differences between protein groups.	No change in hand grip strength (dynamometer), no significant difference between all 4 groups.	SPPB and gait speed (4 m walk test) maintained in protein groups, decreased in control group over time. The 5 chair stand test component increased in time taken for control group but decreased for all protein groups. No differences between protein groups.
Matsuda [52]	24 weeks	Soy protein drink 7.5 g 1/day **7.5 g/day** RT + AE 3/week	(1) BCAA 8 g 1/day **8 g/day** RT + AE 3/week	Skeletal muscle mass (BIA) did not change over time or between groups	Knee extension strength (dynamometer) significantly improved in the soy group but not in the BCAA group. No significant differences between groups. Grip strength (dynamometer) improved in the BCAA group not soy, but no significant differences between groups.	
Roschel [56]	16 weeks	Soy protein 15 g 2/day **30 g/day** RT 2/week	(1) Whey protein 15 g 2/day **30 g/day** RT 2/week (2) Corn Starch 15 g 2/day **30 g/day** RT 2/week	Total and ASMM (DEXA) improved over time but no significant differences between groups. Total fat mass (DEXA) did not change throughout trial in either group.	Hand grip strength (dynamometer) did not change over time in either group, with no significant group differences.	TUG did not change over time or between groups.

Abbreviations: AE, aerobic exercise; ASMM, appendicular skeletal muscle mass; ASMMI, appendicular skeletal muscle mass index; BCAA, branched-chain amino acids; BIA, body composition measured by bioelectrical impedance analysis; BodPod, body composition as measured using air displacement plethysmography; BMD, bone mineral density; BMI, body mass index; CT, computer tomography; DEXA, body composition measured using dual energy X-ray absorptiometry; HRT, hormone replacement therapy; RT, resistance training; SPPB, short physical performance battery; TUG, timed up-and-go. * total daily dose in bold, reported separately to distinguish between per serve amount.

## Data Availability

The data presented in this study are available on request from the corresponding author.

## Supplementary Materials

The following supporting information can be downloaded at: https://www.mdpi.com/article/10.3390/nu15184060/s1, Table S1: Search strategy for Embase database; Table S2: Search strategy for CINAHL database; Table S3: Search strategy for Cochrane Library database; Figure S1: Forest plot of randomized controlled studies examining the effect of plant protein interventions on short physical performance battery (SPPB); Figure S2: Forest plot of randomized controlled studies examining the effect of plant protein interventions on hand grip strength.
