# Supplementary material for: Effects of Plant-Based Protein Interventions, with and without an Exercise Component, on Body Composition, Strength and Physical Function in Older Adults: A Systematic Review and Meta-Analysis of Randomized Controlled Trials
Source: Nutrients. 2023 Sep 19;15(18):4060. doi: 10.3390/nu15184060 (PMC10537483; doi:10.3390/nu15184060)
Supplement: Supplementary file 1 [file nutrients-15-04060-s001.zip › nutrients-2522035-supplementary.pdf]

Supplementary Figures

Study or Subgroup	Plant Protein Mean	Plant Protein SD	Plant Protein Total	Control Mean	Control SD	Control Total	Weight	Mean Difference IV, Random, 95% CI
Beavers 2015	0.1	0.56	12	0.2	0.95	12	27.7%	-0.10 [-0.72, 0.52]
Kreijkamp-Kaspers 2005	0.07	0.9	100	0.18	0.9	102	38.5%	-0.11 [-0.36, 0.14]
Li 2021	-0.12	0.83	31	-0.84	0.86	30	33.8%	0.72 [0.30, 1.14]
**Total (95% CI)**			**143**			**144**	**100.0%**	**0.17 [-0.40, 0.74]**

Heterogeneity: Tau² = 0.20; Chi² = 11.31, df = 2 (P = 0.004); I² = 82%
Test for overall effect: Z = 0.60 (P = 0.55)

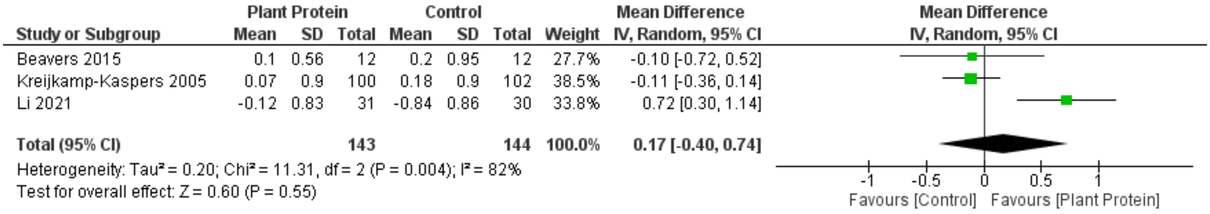

Figure S1. Forest plot of randomized controlled studies examining the effect of plant protein interventions on short physical performance battery (SPPB). Individual study effect estimates (green boxes) and the pooled effect estimate (diamond) are shown. Values are mean differences with 95% confidence intervals (CI) determined using generic inverse-variance random-effects models. Heterogeneity was quantified by I^2 at a significance of p<0.10

Study or Subgroup	Plant Protein Mean	Plant Protein SD	Plant Protein Total	Control Mean	Control SD	Control Total	Weight	Std. Mean Difference IV, Random, 95% CI
Beavers 2015	0.2	9.29	12	-0.9	9	12	11.9%	0.12 [-0.68, 0.92]
Bijeh 2022	1.3	0.34	15	-1.32	2.02	15	11.2%	1.76 [0.90, 2.62]
Imaoka 2019	2.3	7.35	31	2.6	9.19	36	16.3%	-0.04 [-0.52, 0.44]
Imaoka 2022	0	7.96	36	1.4	9.19	36	16.6%	-0.16 [-0.62, 0.30]
Li 2021	0.42	2.06	31	0.49	2.1	30	16.0%	-0.03 [-0.54, 0.47]
Matsuda 2022	0	2	15	2	3	21	13.4%	-0.74 [-1.43, -0.06]
Roschel 2021	0	7.01	22	0	7.01	22	14.7%	0.00 [-0.59, 0.59]
**Total (95% CI)**			**162**			**172**	**100.0%**	**0.07 [-0.35, 0.50]**

Heterogeneity: Tau² = 0.23; Chi² = 21.14, df = 6 (P = 0.002); I² = 72%
Test for overall effect: Z = 0.34 (P = 0.74)

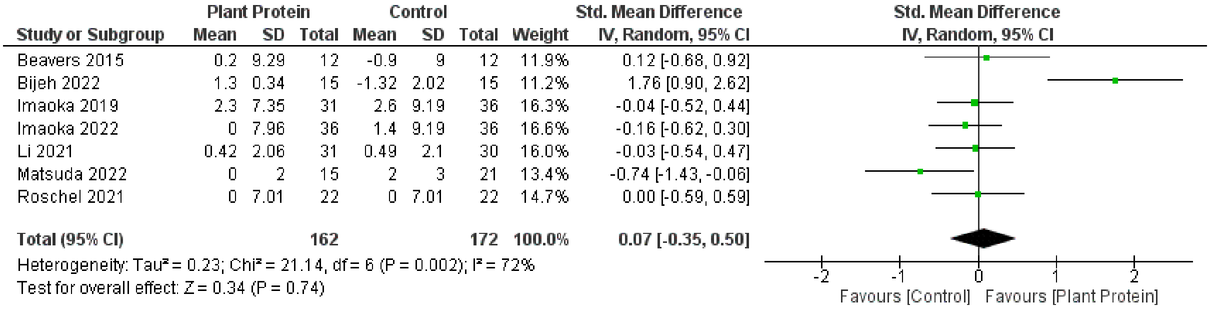

Figure S2. Forest plot of randomized controlled studies examining the effect of plant protein interventions on hand grip strength. Individual study effect estimates (green boxes) and the pooled effect estimate (diamond) are shown. Values are standardised mean differences with 95% confidence intervals (CI) determined using generic inverse-variance random-effects models. Heterogeneity was quantified by I^2 at a significance of p<0.10

Supplementary Tables

Table S1. Search Strategy for Embase Database

Search Line	Search Terms
1	SARCOPENIA/
2	AGED/
3	AGING/
4	"Aged, 80 and over"/
5	FRAIL ELDERLY/
6	FRAILTY/
7	(dynapen* or anabolic resistance).mp.
8	(older adult or older or senior or elder or elderly or ?enarian or geriatric).mp.
9	(sarcop?eni* or aged or aging or frail elderly or frailty).tw.
10	1 or 2 or 3 or 4 or 5 or 6 or 7 or 8 or 9
11	Plant Proteins/
12	Vegetable Proteins/
13	Soybean Proteins/
14	(plant protein* or vegetable protein* or soybean protein*).tw.
15	Soy milk/
16	soy milk.tw.
17	11 or 12 or 13 or 14 or 15 or 16
18	Body Composition/
19	Skeletal Muscle/
20	Adipose Tissue/
21	Bone density/
22	(muscle or muscle mass or fat mass or bone density).mp.
23	18 or 19 or 20 or 21 or 22
24	animals/ not (humans/ and animals/)
25	10 and 17 and 23
26	25 not 24

Table S2. Search Strategy for CINAHL (Cumulative Index to Nursing and Allied Health Literature) Database

Search Line	Search Terms
1	MH sarcopenia
2	MH aging
3	MH frailty syndrome
4	MH frail elderly
5	MH aged
6	TI sarcop#eni* or aging or aged or frail or frail elderly or frailty
7	AB sarcop#eni* or aging or aged or frail or frail elderly or frailty
8	"anabolic resistance" OR dynap#eni* OR "older adult" OR senior OR elder* OR ?enarian OR geriatric
9	S1 OR S2 OR S3 OR S4 OR S5 OR S6 OR S7 OR S8
10	MH plant proteins
11	MH soy proteins
12	MH soy foods
13	TI "plant protein*" or "soy protein*" or "soy food*" or "plant-base*" or "soy milk"
14	AB "plant protein*" or "soy protein*" or "soy food*" or "plant-base*" or "soy milk"
15	MH soy milk
16	S10 OR S11 OR S12 OR S13 OR S14 OR S15
17	S9 AND S16

Table S3. Search Strategy for Cochrane Library Database

Search Line	Search Terms
1	sarcop*enia
2	MeSH descriptor: [Sarcopenia] explode all trees
3	MeSH descriptor: [Aged] explode all trees
4	MeSH descriptor: [Aged, 80 and over] explode all trees
5	MeSH descriptor: [Aging] explode all trees
6	aged or aging
7	dynap*enia
8	"anabolic resistance"
9	"older adult" or older or senior or elderly or *enarian or geriatric
10	MeSH descriptor: [Frail Elderly] explode all trees
11	MeSH descriptor: [Frailty] explode all trees
12	frail or "frail elder" or "frail elderly" or frailty
13	#1 or #2 or #3 or #4 or #5 or #6 or #7 or #8 or #9 or #10 or #11 or #12
14	MeSH descriptor: [Plant Proteins] explode all trees
15	MeSH descriptor: [Plant Proteins, Dietary] explode all trees
16	"plant protein" or "dietary protein" or "vegetable protein" or "plant-base*"
17	MeSH descriptor: [Soy Milk] explode all trees
18	"plant-based protein"
19	MeSH descriptor: [Soy Foods] explode all trees
20	MeSH descriptor: [Soybean Proteins] explode all trees
21	soy
22	"soy milk" or "soy food" or "soybean protein"
23	#14 or #15 or #16 or #17 or #18 or #19 or #20 or #21 or #22
24	#13 and #23 in Trials
